# Supplementary material for: Impact of two of the world's largest protected areas on longline fishery catch rates
Source: Nat Commun. 2020 Feb 20;11:979. doi: 10.1038/s41467-020-14588-3 (PMC7033108; doi:10.1038/s41467-020-14588-3)
Supplement: Supplementary file 1 — Reporting Summary [file 41467_2020_14588_MOESM1_ESM.pdf]

nature research

Corresponding author(s): John Lynham

Last updated by author(s): Dec 6, 2019

Reporting Summary

Nature Research wishes to improve the reproducibility of the work that we publish. This form provides structure for consistency and transparency in reporting. For further information on Nature Research policies, see Authors & Referees and the Editorial Policy Checklist .

Statistics

For all statistical analyses, confirm that the following items are present in the figure legend, table legend, main text, or Methods section.

n/a	Confirmed	
☐	☒	The exact sample size (*n*) for each experimental group/condition, given as a discrete number and unit of measurement
☐	☒	A statement on whether measurements were taken from distinct samples or whether the same sample was measured repeatedly
☐	☒	The statistical test(s) used AND whether they are one- or two-sided<br>*Only common tests should be described solely by name; describe more complex techniques in the Methods section.*
☐	☒	A description of all covariates tested
☐	☒	A description of any assumptions or corrections, such as tests of normality and adjustment for multiple comparisons
☐	☒	A full description of the statistical parameters including central tendency (e.g. means) or other basic estimates (e.g. regression coefficient) AND variation (e.g. standard deviation) or associated estimates of uncertainty (e.g. confidence intervals)
☐	☒	For null hypothesis testing, the test statistic (e.g. *F*, *t*, *r*) with confidence intervals, effect sizes, degrees of freedom and *P* value noted<br>*Give P values as exact values whenever suitable.*
☒	☐	For Bayesian analysis, information on the choice of priors and Markov chain Monte Carlo settings
☒	☐	For hierarchical and complex designs, identification of the appropriate level for tests and full reporting of outcomes
☐	☒	Estimates of effect sizes (e.g. Cohen's *d*, Pearson's *r*), indicating how they were calculated

Our web collection on statistics for biologists contains articles on many of the points above.

Software and code

Policy information about availability of computer code

Data collection	No software was used to collect the data. It was either downloaded from publicly available sources online or transferred to JL using a standard file transfer protocol.
Data analysis	The majority of the analysis was performed using R Version 3.6.0 and the IDE RStudio (Version 1.2.1335). Some initial data manipulation and one set of regressions were performed using STATA Version 15.

For manuscripts utilizing custom algorithms or software that are central to the research but not yet described in published literature, software must be made available to editors/reviewers. We strongly encourage code deposition in a community repository (e.g. GitHub). See the Nature Research guidelines for submitting code & software for further information.

Data

Policy information about availability of data

All manuscripts must include a data availability statement. This statement should provide the following information, where applicable:

- Accession codes, unique identifiers, or web links for publicly available datasets
- A list of figures that have associated raw data
- A description of any restrictions on data availability

The aggregate logbook data that support the findings of this study are available from NOAA Fisheries (https://www.fisheries.noaa.gov/resource/data/hawaii-longline-fishery-logbook-summary-reports). The Observer Program data that support the findings of this study are available from NOAA Fisheries (https://inport.nmfs.noaa.gov/inport/item/21854) but restrictions apply to the availability of these data, which contain business confidential information. Under the terms of a non-disclosure agreement with NOAA, JL can not make these data publicly available. The AIS vessel location data that support the findings of this study are available from Global Fishing Watch (https://globalfishingwatch.org/). Under the terms of a data-sharing agreement with GFW, JL can not make these data publicly available.

The source data underlying Figs 2 and 3 are provided as a Source Data file. The source data underlying Figs 1b and 4 are not publicly available according to the non-

disclosure agreement with NOAA, described above. Summary data used to create Figures 1b and 4 are provided in the Source Data file and the exact code used to create these summaries from the source data is also provided (see Code Availability section).

Field-specific reporting

Please select the one below that is the best fit for your research. If you are not sure, read the appropriate sections before making your selection.

☐ Life sciences ☒ Behavioural & social sciences ☐ Ecological, evolutionary & environmental sciences

For a reference copy of the document with all sections, see nature.com/documents/nr-reporting-summary-flat.pdf

Behavioural & social sciences study design

All studies must disclose on these points even when the disclosure is negative.

Study description

Data are quantitative, primarily observer data recording the time, date, location and fish caught during a fishing event ("set").

Research sample

The research sample is federal records of fishing events in the Hawaii and American Samoa longline fisheries. The data collection is mandated as part of fisheries management regulations. A random 20% of tuna fishing trips and 100% of swordfish trips have a NOAA observer on board who collects a variety of scientific information on the fishing trip. In particular, the information we use is the date of a fishing event, its location, and how many fish of different species were caught. There is a unique anonymous indicator for each fishing vessel, allowing us to observe the same vessel over time.

Sampling strategy

We used all of the data provided to us but we restricted the sample to 2010-2017 to address possible concerns about bias due to impacts on fish markets and fishing behavior during the Great Recession. We have run all of our analysis using the full sample (1994-2017) and the results are qualitatively the same.

Data collection

We are not the primary data collectors, it was provided to us by the NOAA Fishery Observer Program. The Hawaii-based longline fishery targeting tuna and swordfish has been monitored under a mandatory observer program since February 1994. In 2017, there was a total of 145 vessels active in this fishery. The American Samoa-based longline fishery has been monitored under a similar program since April 2006. In 2016, there was a total of 20 vessels active in this fishery. Beginning in the year 2000, the Hawaii observer program significantly increased its observer coverage. In the period March 1994 to September 2000, 322 observer trips were completed, averaging 46 trips per calendar year from 1994 to 1999. From October 2000 to September 2001, 234 observer trips were completed, representing over a 500% increase from that in the previous years. Observers document interactions with protected species, identify and enumerate fish catches and bycatch, and collect various requested samples for life history studies. Observers are NOAA employees and are not affiliated with the owners, captains, or crew of any of the fishing vessels. All pelagic longline fishing trips are required to have a fishery observer on board if requested by NOAA Fisheries. For the Hawaii shallow-set longline fishery (i.e. for vessels targeting swordfish), NOAA places observers on every single fishing trip. The deep-set longline fisheries targeting tunas in Hawaii and American Samoa, on the other hand, have approximately 20% observer coverage of all trips (we refer to these fisheries as tuna fisheries). As a result, this dataset contains the location of every fishing event (longline set) for 100% of swordfish trips and a quasi-random 20% of tuna trips since 2000 (although we restrict our attention to trips that began on or after January 1st 2010). We use the term quasi-random since we have been told that although every effort is made to randomly assign observers to tuna trips, this is not always possible based on observer availability and the timing of fishing trips.

Global Fishing Watch is a non-profit organization and website launched in September 2016 by Google in partnership with Oceana and SkyTruth to provide the world's first global view of commercial fishing activities. At any moment, 200,000 vessels are publicizing their locations via a system known as the Automatic Identification System (AIS). AIS is intended, primarily, to allow ships to view marine traffic in their area and to be seen by that traffic. This requires a dedicated VHF (Very High Frequency) AIS transceiver that automatically broadcasts information about a vessel, such as its position, speed, navigational status, name, and VHF call sign.
This information is broadcast at regular intervals, in some cases, as frequently as every fifteen seconds. Vessels fitted with AIS transceivers can be tracked by other ships, by AIS base stations located along coast lines or, when out of range of terrestrial networks, by a growing number of satellites that are fitted with special AIS receivers. The International Maritime Organization's International Convention for the Safety of Life at Sea requires AIS to be fitted aboard international voyaging ships with 300 or more gross tonnage, and for all passenger ships regardless of size. The US Coast Guard now requires it for all vessels larger than 65 feet. Despite its widespread use, AIS information typically supplements marine radar, which continues to be the primary method of collision avoidance for water transport.

Global Fishing Watch extracts the AIS tracks for fishing vessels and enables users with Internet access to monitor fishing activity globally, and to view individual vessel tracks. They also partner with academic researchers to provide more fine-scale data. We requested and obtained individual fishing tracks for all of the vessels that we could identify within the Hawaii and American Samoa longline fisheries. We obtained tracks for 148 different vessels (identified by their MMSI: Maritime Mobile Service Identity number) but a number of these vessels had yet to emit a signal leaving a total of 128 vessels with observed tracks. Initially, the dataset contains 5,592,202 observations of vessel locations ranging from January 1st 2015 to December 31st 2017.

Timing

Observer data: Jan 1st 2010 to December 31st 2017
GFW data: January 1st 2015 to December 31st 2017

Data exclusions

Observer Data prior to 2010 were excluded. This was pre-planned. Including this data does not change our conclusions. The policy changes we study occurred in 2014 and 2016.

Non-participation	None. Vessels are legally required to have an observer onboard if randomly selected.
Randomization	Treatment status was not randomized. As a result, we use a differences-in-differences methodology to account for the possible influence of unobserved confounds on the treated fishing vessels.<br><br>In order to make credible statements about the causal impacts of the expansions, we need to find a control for the Hawaii tuna fishery that is influenced by the same unobserved factors that might be correlated with the monument expansions (such as changes in oceanographic conditions) but this control is unaffected by the expansions themselves, thus satisfying the excludability and no interference assumptions. We believe we have novel and appropriate counterfactuals for both expansions: bycatch of bigeye and yellowfin tuna in the Hawaii longline swordfish fleet for the PRI expansion and incidental catch of bigeye and yellowfin tuna in the American Samoa longline albacore tuna fleet for PMNM (the dashed grey lines in Figure 2 of the main article show the mean of the four CPUE measures for these counterfactual fisheries).<br><br>The Hawaii swordfish fleet is a small fleet that mainly fishes to the north and east of Honolulu since these are the best grounds for swordfish (Figure 1A in main article). PRI lies to the south and west of Honolulu. This fleet has 100% observer coverage due to their high bycatch rates of protected species. Since 1994, a swordfish set has never been recorded inside PRI by NOAA observers: thus the Hawaii swordfish fleet should be unaffected by the PRI expansion. Critically, the swordfish fleet unintentionally catches bigeye and yellowfin tuna when targeting swordfish. Any environmental variation that is influencing abundance of these tuna species in the Pacific should be reflected in their bycatch rates, thus allowing us to control for or ``exclude'' environmental variation as a source of bias.<br>The current scientific consensus is that bigeye tuna is one large population spread across the Pacific Ocean and yellowfin tuna is three or more large populations. Furthermore, the swordfish fleet is US-flagged, subject to roughly the same regulations as the Hawaii tuna fleet, and sells their catch at the exact same auction. This should allow us to exclude unobserved changes in regulatory, institutional, and market conditions as potential sources of bias in our regression estimates.<br><br>An American Samoa longline permit gives the right to fish around American Samoa, Guam, the Northern Mariana Islands, and the PRI areas, but it has never included the legal right to fish in the northwestern Hawaiian islands (Figure 1A in the main article shows the spatial extent of this fleet): thus the American Samoa tuna fleet should be unaffected by the expansion of PMNM.<br>Again, this fleet is primarily targeting a different species, albacore tuna, so their incidental catch of bigeye and yellowfin tuna serves as a random sampling of their abundance.<br>In the SOM, we show that catch of bigeye and yellowfin is strongly positively correlated across all three fisheries, confirming our claim that they are plausible counterfactuals. It can be seen in the SOM and in Figure 2 in the main article that the timing of both expansions appears to coincide with a general increase in bigeye and yellowfin tuna abundance; this has been linked to favorable recruitment conditions in 2012.<br>It is important to note that NOAA observers record every fish caught, whether it is a target species or not. Thus, we have a very accurate measure of trends in bigeye and yellowfin tuna abundance that should be unaffected by the monument expansions. The American Samoa tuna fleet is also US-flagged and subject to similar regulations to the Hawaii tuna fleet.

Reporting for specific materials, systems and methods

We require information from authors about some types of materials, experimental systems and methods used in many studies. Here, indicate whether each material, system or method listed is relevant to your study. If you are not sure if a list item applies to your research, read the appropriate section before selecting a response.

Materials & experimental systems

n/a	Involved in the study
☒	☐ Antibodies
☒	☐ Eukaryotic cell lines
☒	☐ Palaeontology
☒	☐ Animals and other organisms
☒	☐ Human research participants
☒	☐ Clinical data

Methods

n/a	Involved in the study
☒	☐ ChIP-seq
☒	☐ Flow cytometry
☒	☐ MRI-based neuroimaging
