# Supplementary material for: Impact of two of the world's largest protected areas on longline fishery catch rates
Source: Nat Commun. 2020 Feb 20;11:979. doi: 10.1038/s41467-020-14588-3 (PMC7033108; doi:10.1038/s41467-020-14588-3)
Supplement: Supplementary file 3 — Supplementary Information [file 41467_2020_14588_MOESM3_ESM.pdf]

Supplementary Information

Impact of two of the world's largest protected areas on longline fishery catch rates

(Lynham et al.)

Supplementary Discussion

Additional Background Information on the Monuments and the Fishery

On June 15, 2006, President George W. Bush signed Proclamation 8031, designating the waters of the Northwestern Hawaiian Islands as a national monument (Papahānaumokuākea Marine National Monument) under the 1906 Antiquities Act. This created a protected area of 363,000 square kilometers. It's important to note that since 1991, NOAA has banned longline fishing within 50 nautical miles of the Northwestern Hawaiian Islands so that when the monument was declared, the only fishing taking place was bottom fishing by less than ten boats. Then, on January 6, 2009, President Bush proclaimed the Pacific Remote Islands Marine National Monument a national monument, creating a second large and remote protected area in the Pacific Ocean (225,000 square kilometers). Both of these monuments protected the waters close to land and did not pose a major threat to pelagic longline fisheries. Our analysis in this paper focuses on the subsequent expansions of these monuments by President Barack Obama, which have been more controversial. On September 25, 2014, President Obama more than quintupled the size of PRI by closing an additional 1,054,000 square kilometers to all fishing and extractive activities. The monument now protects 1,270,000 square kilometers of ocean space (an area larger than the land mass of South Africa). Then, on August 26, 2016, President Obama quadrupled the size of Papahānaumokuākea Marine National Monument to 1,510,000 square kilometers. It is currently the third largest protected area in the world. Supplementary Table 1 summarizes the main Presidential proclamations and the cumulative area protected within each monument. We take the dates of the signing of the proclamations by President Obama as the start dates of the expansions since the prohibitions in a proclamation are supposed to take effect immediately.[1]

Supplementary Table 1: Timeline of Events

Date	Event	Cumulative Monument Area (square kilometers)
June 15, 2006	President Bush creates PMNM	PMNM=363,000
January 6, 2009	President Bush creates PRI	PRI=225,000
September 25, 2014	President Obama expands PRI	PRI=1,270,000
August 26, 2016	President Obama expands PMNM	PMNM=1,510,000

The primary fishing fleet impacted by the expansions of the two Pacific marine monuments is the limited-entry Hawaii-based longline fleet. There is also a small bottomfish fleet that occasionally fished within the monument waters that we do not include in our analysis. Also, as mentioned in the main text, due to data limitations, we have not analyzed the impact of the PRI expansions on the American Samoa-based purse seine fleet, which used to fish within PRI. However, given that the Western Pacific Regional Fishery Management Council state in their 2013 Annual Report that the "combined amount of fish harvested from these areas from the U.S. purse seine on average is less than 5 percent of their total annual harvest", this suggests that the impacts would be fairly small.

The Hawaii-based longline fleet is a relatively large industrialized fishery with around 145 permitted vessels with lengths exceeding 65 feet. Total annual gross revenues are in excess of $100 million and total catch exceeds 37 million pounds. Most fish are landed at the Honolulu Fish Auction and then sold domestically or exported. This fishery is the largest commercial fishing sector in the Hawaiian Islands. The fishery is composed of three distinct ethnic groups: Vietnamese Americans, European Americans, and Korean Americans. There tends to be strong information-sharing and business ties within each ethnic group.[2]

The fleet is divided into two sub-fleets: the deep-set tuna fleet and the shallow-set swordfish fleet. Deep and shallow refer to the depths at which the long lines of hooks are set. Shallow sets tend to have higher rates of turtle, marine mammal, and shark bycatch. The tuna fleet primarily target bigeye tuna (*Thunnus obesus*) but also yellowfin tuna (*Thunnus albacares*). The swordfish fleet targets swordfish (*Xiphias gladius*). For tuna, trips typically last 14 to 30 days. Trips in the swordfish fishery typically last 22 to 35 days. Although the tuna fishery has been regulated with a Total Allowable Catch on bigeye tuna since 2009, the fleet has been allowed to exceed this by transferring unused TAC

allocation from other US territories.[3;4] Nevertheless, there are some years when the area regulated by the Western and Central Pacific Fisheries Commission (WCPFC) has been temporarily closed to Hawaii-based vessels. This ranges from 0 days out of the year from 2011-2014 to a maximum of 65 days in 2015.[4] The area managed by the Inter-American Tropical Tuna Commission (IATTC) has also occasionally prevented vessels from retaining bigeye tuna once catch limits have been reached but these limits typically only apply to vessels greater than 24m in length, which only constitutes about 25% of the fleet.[4] In all of our regression analysis that includes additional control variables, we include dummy variables to indicate whether the WCPFC or IATTC region is closed on a particular day.

In the swordfish fishery, bycatch caps have been set for Loggerhead and Leatherback sea turtles. There used to be a limit on swordfish sets per year (2,120) but this was removed in 2009. There is no limit on total catch. Some vessels switch back-and-forth between both sub-fleets but participation is generally stable within a particular sub-fleet. The swordfish fleet is quite small and has been declining over time (in part due to increasingly strict regulations) whereas the tuna fleet is relatively robust and stable. For example, longline vessels made 1,563 trips in 2017, of which a record 1,502 were deep-set trips and only 61 were shallow-set trips. Further, deep-set trips are increasing over time while the shallow-set trips are on a slow decline. The deep-set longline fishery produced $99.1 million in revenue in 2016. This fishery represented 88% of the total revenue for pelagic fish in Hawaii in 2016. The shallow-set longline fishery produced $2.5 million in revenue which accounted for only 2% of the total revenue. The remaining revenue for pelagic fish in Hawaii came from troll and handline fisheries in the main Hawaiian islands. A very small percentage of revenue (less than 1%) came from the offshore handline fishery.

Total Catch and Revenue

In Figure 2 in the main text, we plot total annual catch of bigeye tuna (the primary target species), other tuna species (including yellowfin), and swordfish, from 2010 to 2017 for all Hawaii-based pelagic longline fishing trips. It is very clear from the figure that total catch has not declined since the 2014 and 2016 expansions. The four years with the highest total catch since 2010 are the four years since the expansions began (2014-2017).

In Figure 3 in the main text, we plot total annual catch per unit effort of bigeye tuna, other tuna species, and swordfish, from 2010 to 2017 for all Hawaii-based pelagic longline fishing trips. The three measures of effort we use are total hooks, total fishing sets, and total fishing trips. It is clear that total catch per unit of fishing effort has not declined since the 2014 and 2016 expansions.

As suggested by the general upward trend in catch (35% increase), we are not surprised to observe that total revenue in the fishery has generally increased since the expansions began in 2014 (see Supplementary Figure 1). In fact, total revenue increased 39.6% from 2010 to 2017 and average revenue during 2014-2017 was 13.7% higher than in 2010-2013. It appears fairly clear that the two monument expansions are not correlated with a drop in total revenue in this fishery. Although the fishery as a whole brought in more money in 2016 and 2017 than in any previous year, one important caveat is that we do not have detailed data on catch and revenues at the individual vessel level. Although the total number of vessels has remained relatively constant over this time period, it could very easily be the case that individual vessels have experienced a decline in catch or total revenue since the monuments expanded.

Supplementary Figure 1: Total Revenue from Commercial Pelagic Fisheries (1,000s of $)

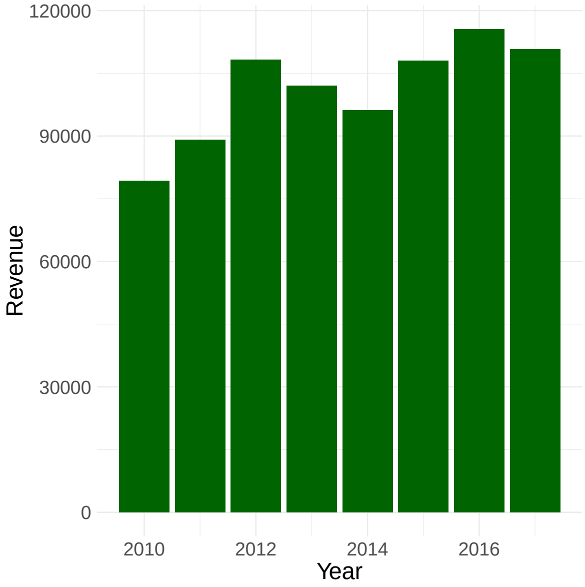

Source: Western Pacific Regional Fishery Management Council Pelagic Fisheries of the Western Region Annual Reports from 2011 to 2017.

Supplementary Methods

Catch and CPUE Regressions

We now turn to a more detailed micro-level analysis of changes to the fleet following the expansions. We start by estimating regressions of the following form:

$$y_{i,t} = \beta_0 + \beta_1 \text{PRI}_t + \beta_2 \text{PMNM}_t + \mathbf{m_t'}\mu + \mathbf{v_i'}\phi + \mathbf{X_{i,t}'}\chi + u_{i,t} \tag{1}$$

where $y_{i,t}$ is the outcome variable of interest (such as catch per unit of effort or distance traveled) for vessel i in time period t (typically a day). β_0 is the standard intercept term and β_1 and β_2 are the main slope parameters of interest. PRI_t is a dummy variable that takes the value of 0 for all dates prior to the PRI expansion and the value of 1 for all dates including and following the expansion date. The same logic applies to PMNM_t. $\mathbf{m_t}$ is a vector of month dummies, $\mathbf{v_i}$ is a vector of individual vessel dummies, and $\mathbf{X_{i,t}}$ is a vector of additional controls (oceanographic conditions, experimental fishing sets, etc.). Although we are indexing $\mathbf{X_{i,t}}$ with i and t, not all of these variables will vary across vessels or across time. The specific controls we include are the following: an indicator dummy for whether the Western Pacific region was closed due to the fleet reaching its TAC for that region (depending on the year this ranges from 0 to 31% of the days in the year), an indicator dummy for whether the Eastern region of the Pacific was closed to vessels over 24m in length due to a binding TAC (75% of Hawaii-based vessels are less than 24m and these closures ranged from 0 to 16% of the days in a year), an indicator variable for whether there was experimental research also being conducted as part of the fishing set (sometimes researchers request NOAA observers to do additional tasks, for example, some trips conducted research on whale deterrents, different types of hooks, weighted gear, and shark survival - experimental sets comprise less than 4% of all sets in the dataset), the monthly Nino 3.4 index provided by the National Center for Atmospheric Research[5], the same Nino 3.4 index lagged by one year, the Nino 3.4 index lagged by two years, and, finally, the Nino 3.4 index lagged by three years. The Nino 3.4 anomalies may be thought of as representing the average equatorial sea surface temperatures across the Pacific from about the dateline to the South American coast. The Nino 3.4 index typically uses a 5-month running mean, and El Nino or La Nina events are defined

when the Nino 3.4 sea surface temperatures exceed +/- 0.4C for a period of six months or more. We estimate the slope coefficients in each regression using ordinary least squares estimation and we report heteroskedasticity-robust standard errors in all tables. The main dataset we use for our analysis is described in the next paragraphs.

The Hawaii-based longline fishery targeting tuna and swordfish has been monitored under a mandatory observer program since February 1994. In 2017, there was a total of 145 vessels active in this fishery. The American Samoa-based longline fishery has been monitored under a similar program since April 2006. In 2016, there was a total of 20 vessels active in this fishery. Beginning in the year 2000, the Hawaii observer program significantly increased its observer coverage. In the period March 1994 to September 2000, 322 observer trips were completed, averaging 46 trips per calendar year from 1994 to 1999. From October 2000 to September 2001, 234 observer trips were completed, representing over a 500% increase from that in the previous years. Observers document interactions with protected species, identify and enumerate fish catches and bycatch, and collect various requested samples for life history studies. Observers are NOAA employees and are not affiliated with the owners, captains, or crew of any of the fishing vessels. All pelagic longline fishing trips are required to have a fishery observer on board if requested by NOAA Fisheries. For the Hawaii shallow-set longline fishery (i.e. for vessels targeting swordfish), NOAA places observers on every single fishing trip. The deep-set longline fisheries targeting tunas in Hawaii and American Samoa, on the other hand, have approximately 20% observer coverage of all trips (we refer to these fisheries as tuna fisheries). As a result, this dataset contains the location of every fishing event (longline set) for 100% of swordfish trips and a quasi-random 20% of tuna trips since 2000 (although we restrict our attention to trips that began on or after January 1st 2010). We use the term quasi-random since we have been told that although every effort is made to randomly assign observers to tuna trips, this is not always possible based on observer availability and the timing of fishing trips. Vessels are allowed to switch back and forth from targeting tuna and swordfish but this is not allowed during a trip (and would be costly to do in terms of adjusting gear so that the lines sit shallower or deeper in the water). Although swordfish vessels will sometimes go on tuna trips, the reverse is quite rare. For example, since 2010, 76% of the vessels in the observer dataset are only observed taking tuna trips. The remaining 24% are split evenly between majority tuna and majority swordfish trips. No vessel has exclusively targeted swordfish since 2010 but 6 vessels are observed with 80% or more swordfish sets. Thus, the observer dataset has good temporal coverage of the fleet but not complete cross-sectional coverage of the fleet. Another drawback is that we only observe the location of fishing events, we don't know where else the vessel has traveled (i.e. searching for fish) and when exactly a trip begins and ends (but we are able to approximate this).

We focus our attention on tuna trips departing from Hawaii, since this fleet composes the bulk of the fishery in terms of effort and revenue. We define catch as the total number of bigeye and yellowfin tuna caught: observers count but do not weigh the fish caught. We use two different measures of aggregate catch: total annual catch and total monthly catch. We use four different measures of catch per unit effort: catch-per-fishing-trip, catch-per-fishing-set, catch-per-1,000-hooks-set, and catch-per-kilometer-traveled. Results from estimating different versions of Equation 1 with the two catch measures are shown in Supplementary Table 2, and results using the four different catch-per-unit effort variables are shown in Table 1 and Supplementary Tables 3, 4, and 5. It can be seen that both expansions tend to be correlated with an *increase* in catch and catch-per-unit-effort. The efficiency of a unit of fishing effort does not appear to be negatively impacted by the expansions. We sometimes observe a loss of statistical significance or switches in the sign of the estimated coefficients when we include year fixed effects. This is not too surprising when we consider that the monument dummies (especially PMNM) are almost perfectly collinear with the year dummies. For example, the Pearson correlation between the PMNM dummy and the 2017 dummy is 0.85. In practical terms, this means that the effect of the PMNM expansion is being estimated off of the last three months of 2016 instead of using variation from all 15 months that the monument expansion was in effect in the sample (October 2016 to December 2017).

Supplementary Table 2: Total Catch of Bigeye and Yellowfin Tuna

	(1)	(2)	(3)	(4)	(5)
Constant	44,002.600***	3,574.877***	4,104.993***	3,539.848***	−14,668.960
	(3,190.623)	(176.085)	(287.197)	(379.403)	(14,745.050)
PRI Expansion	14,673.900***	1,238.818***	1,201.416***	−51.857	−414.623
	(4,051.426)	(285.135)	(257.504)	(759.598)	(788.050)
PMNM Expansion	8,958.500***	843.679**	675.810**	−316.503	−539.231
	(2,496.794)	(409.008)	(330.183)	(811.179)	(985.255)
Month Dummies	No	No	Yes	Yes	Yes
Year Dummies	No	No	No	Yes	Yes
El Nino Controls	No	No	No	No	Yes
Observations	8	96	96	96	96
R^2	0.785	0.299	0.524	0.588	0.612

Notes: The dependent variable in Column (1) is total annual catch. The dependent variable in Columns (2)-(5) is total monthly catch. Each regression tests whether catch increases following the first expansion, and again following the second expansion. The sample runs from January 1st 2010 to December 31st 2017. Heteroskedasticity-robust standard errors presented in parentheses. *p<0.1; **p<0.05; ***p<0.01

Supplementary Table 3: Catch of Bigeye and Yellowfin Tuna per Set

	(1)	(2)	(3)	(4)	(5)
Constant	11.708***	13.289***	11.792***	8.925***	0.418
	(0.093)	(0.202)	(0.265)	(0.702)	(10.923)
PRI Expansion	3.402***	3.344***	−0.195	−0.413	−1.623***
	(0.173)	(0.173)	(0.519)	(0.527)	(0.553)
PMNM Expansion	2.827***	2.560***	1.212**	1.466***	0.622
	(0.241)	(0.239)	(0.475)	(0.479)	(0.637)
Month Dummies	No	Yes	Yes	Yes	Yes
Year Dummies	No	No	Yes	Yes	Yes
Vessel Dummies	No	No	No	Yes	Yes
Additional Controls	No	No	No	No	Yes
Observations	29,750	29,750	29,750	29,750	29,750
R^2	0.036	0.056	0.061	0.112	0.117

Notes: Each successive column adds additional controls to a simple regression test of whether CPUE increases following the first expansion and again following the second expansion (Column (1)). The sample runs from January 1st 2010 to December 31st 2017. Heteroskedasticity-robust standard errors presented in parentheses. The Additional Controls are whether the set included an experimental component, a dummy variable for whether the WCPFC waters were closed to fishing, a dummy variable for whether IATTC waters were closed to vessels longer than 24m, Monthly El Nino indicator, Monthly El Nino indicator lagged by one year, Monthly El Nino indicator lagged by two years, and Monthly El Nino indicator lagged by three years. *p<0.1; **p<0.05; ***p<0.01

Supplementary Table 4: Catch of Bigeye and Yellowfin Tuna per Trip

	(1)	(2)	(3)	(4)	(5)
Constant	163.345***	180.774***	160.848***	124.392***	52.468
	(2.847)	(6.375)	(8.108)	(18.204)	(298.964)
PRI Expansion	37.844***	36.553***	−12.392	−18.574	−30.842**
	(5.055)	(4.948)	(15.022)	(13.971)	(14.517)
PMNM Expansion	32.410***	30.649***	34.221**	37.421***	36.269**
	(7.362)	(7.050)	(14.708)	(13.084)	(17.048)
Month Dummies	No	Yes	Yes	Yes	Yes
Year Dummies	No	No	Yes	Yes	Yes
Vessel Dummies	No	No	No	Yes	Yes
Additional Controls	No	No	No	No	Yes
Observations	2,155	2,155	2,155	2,155	2,155
R^2	0.065	0.123	0.142	0.374	0.385

Notes: Each successive column adds additional controls to a simple regression test of whether CPUE increases following the first expansion and again following the second expansion (Column (1)). The sample runs from January 1st 2010 to December 31st 2017. Heteroskedasticity-robust standard errors presented in parentheses. The Additional Controls are whether the set included an experimental component, a dummy variable for whether the WCPFC waters were closed to fishing, a dummy variable for whether IATTC waters were closed to vessels longer than 24m, Monthly El Nino indicator, Monthly El Nino indicator lagged by one year, Monthly El Nino indicator lagged by two years, and Monthly El Nino indicator lagged by three years. *p<0.1; **p<0.05; ***p<0.01

Supplementary Table 5: Catch of Bigeye and Yellowfin Tuna per Kilometer Traveled

	(1)	(2)	(3)	(4)	(5)
Constant	0.072***	0.099***	0.086***	0.056***	−0.165
	(0.002)	(0.005)	(0.006)	(0.010)	(0.238)
PRI Expansion	0.022***	0.019***	0.003	−0.001	−0.011
	(0.003)	(0.003)	(0.010)	(0.010)	(0.010)
PMNM Expansion	0.017***	0.017***	0.005	0.006	−0.013
	(0.005)	(0.005)	(0.008)	(0.008)	(0.012)
Month Dummies	No	Yes	Yes	Yes	Yes
Year Dummies	No	No	Yes	Yes	Yes
Vessel Dummies	No	No	No	Yes	Yes
Additional Controls	No	No	No	No	Yes
Observations	2,155	2,155	2,155	2,155	2,155
R^2	0.054	0.224	0.233	0.339	0.357

Notes: Each successive column adds additional controls to a simple regression test of whether CPUE increases following the first expansion and again following the second expansion (Column (1)). The sample runs from January 1st 2010 to December 31st 2017. Heteroskedasticity-robust standard errors presented in parentheses. The Additional Controls are whether the set included an experimental component, a dummy variable for whether the WCPFC waters were closed to fishing, a dummy variable for whether IATTC waters were closed to vessels longer than 24m, Monthly El Nino indicator, Monthly El Nino indicator lagged by one year, Monthly El Nino indicator lagged by two years, and Monthly El Nino indicator lagged by three years. *p<0.1; **p<0.05; ***p<0.01

Distance

If catch and traditional measures of CPUE have not declined, it may still be the case that the monuments are causing vessels to exert more effort to catch the same quantity of fish as before (particularly if effort is expanding along dimensions other than the number of fishing sets or hooks used). An obvious example is distance traveled. Unfortunately, total trip distance is not reported in the observer dataset but we are able to obtain a decent approximation. We observe the date and geographical coordinates of all sets on fishing trips with an observer on-board. We restrict our sample to trips that began in 2010 or later and this leaves around 39,700 sets. We also initially drop 13 observations from the original dataset of 90,860 because they were missing either latitude or longitude coordinates. Nine of these 13 observations are from before 2010. The remaining four observations are missing dates (these are the only observations with missing dates). To estimate trip distance, we calculate the distance covered by the sequence of sets on a trip plus the distances from both starting and ending sets to the Honolulu Fish Auction (or to the main port in Pago Pago for trips departing from American Samoa). We drop any trips that have a starting set in December 2017 to avoid underestimating the distance of these trips (since some may have continued into January 2018, which is not included in the dataset we obtained). Including December 2017 trips would most likely bias our results in favor of finding zero or positive impacts of the expansions on trip distance.

Supplementary Figure 2 shows the mean trip distance per month from 2010 to 2017. Supplementary Figure 3 shows the total trip distance per month. Visually, there does not appear to be much evidence that either expansion is correlated with the tuna fleet traveling greater distances. In fact, it appears that both the average trip distance and total kilometers traveled have decreased slightly since the expansions began. The two months with the shortest mean trip distance are following the PRI expansion. Comparing the time periods prior to and following the PRI expansion in Supplementary Figure 3, it appears that there is an increasing trend prior to the expansions that is then halted following the PRI expansion. Regression analysis using the model in Equation 1 is presented in Supplementary Table 6 and confirms that there is no statistical increase in distance traveled after the expansion. Column (1) of Supplementary Table 6 presents the results from regressing trip distance (measured in kilometers) on a constant (intercept term), a dummy variable for the PRI expansion, and a dummy variable for the PMNM expansion. The coefficient on the PRI dummy variable is *negative* and statistically significant at the 1% level. This demonstrates that trip distances have been shorter on average since President Obama expanded the PRI monument in September 2014. Thus, we can confidently reject the hypothesis that either expansion is correlated with the fleet traveling further than before. Columns (2), (3), and (4) present results from running the same regression but with month, year, and vessel fixed effects (included as separate dummy variables). The qualitative results are unchanged but weaker in terms of statistical significance. Column (5) includes additional controls. The earlier caveat about interpreting the coefficient on the PMNM expansion when including year fixed effects is also relevant here.

Supplementary Figure 2: Mean Trip Distance by Month for Hawaii Tuna Trips

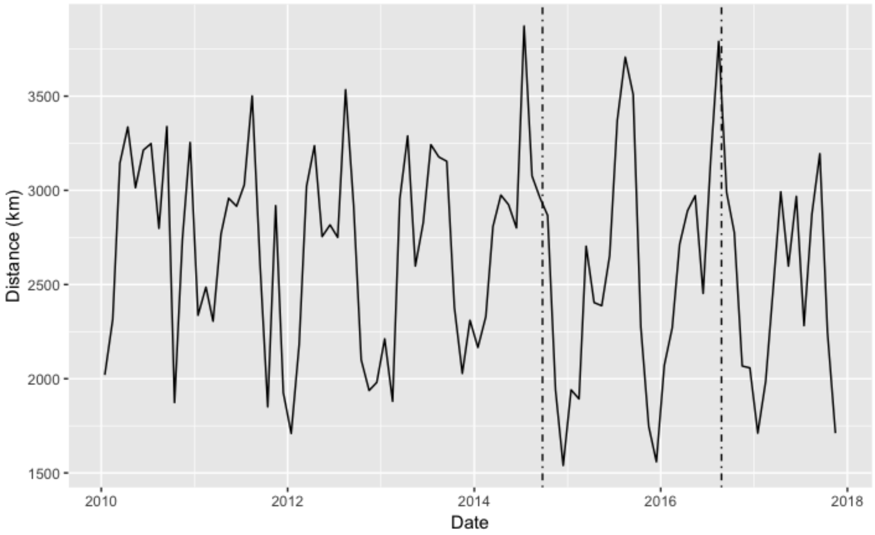

Notes: This shows the mean trip distance by month for all trips targeting tuna with a starting set on or after January 1st 2010 and before December 1st 2017. Vertical dashed lines indicate the dates of the two monument expansions. First line is September 25, 2014. The second line is August 26, 2016.

Supplementary Figure 3: Total Distance Traveled on Hawaii Tuna Trips by Month

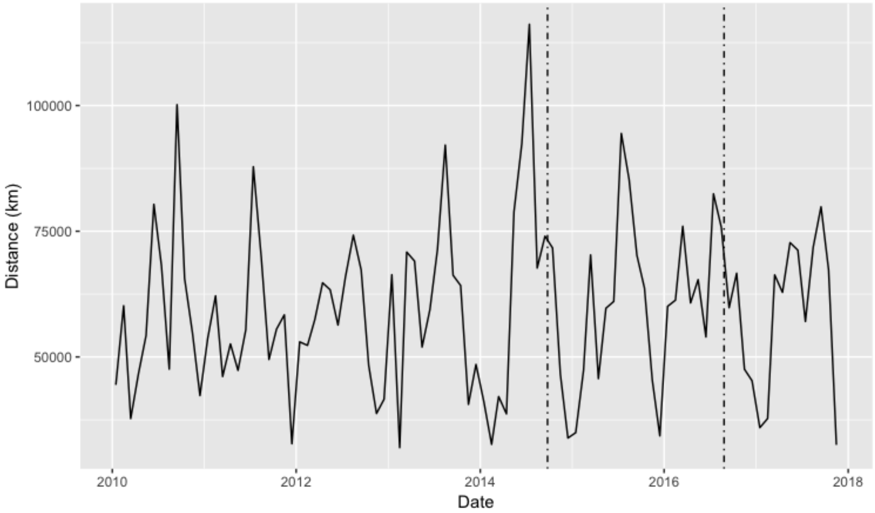

Notes: This shows the total kilometers traveled by month for all trips targeting tuna with a starting set on or after January 1st 2010 and before December 1st 2017. Vertical dashed lines indicate the dates of the two monument expansions. First line is September 25, 2014. The second line is August 26, 2016.

Supplementary Table 6: Distance Traveled

	(1)	(2)	(3)	(4)	(5)
Constant	2,696.761***	2,057.894***	2,685.587***	2,752.639***	2,134.089
	(29.922)	(53.035)	(198.016)	(202.571)	(2,648.118)
PRI Expansion	−160.487***	−106.739**	−95.113*	−137.216	−70.356
	(57.078)	(51.681)	(49.162)	(126.486)	(128.033)
PMNM Expansion	−54.504	−79.293	−89.489	168.381	471.314***
	(74.432)	(69.182)	(64.357)	(129.039)	(167.945)
Month Dummies	No	Yes	Yes	Yes	Yes
Year Dummies	No	No	Yes	Yes	Yes
Vessel Dummies	No	No	No	Yes	Yes
Additional Controls	No	No	No	No	Yes
Observations	2,155	2,155	2,155	2,155	2,155
R^2	0.007	0.177	0.407	0.411	0.440

Notes: Each successive column adds additional controls to a simple regression test of whether distance traveled increases following the first expansion and again following the second expansion (Column (1)). The sample runs from January 1st 2010 to December 31st 2017. Heteroskedasticity-robust standard errors presented in parentheses. The Additional Controls are whether the set included an experimental component, a dummy variable for whether the WCPFC waters were closed to fishing, a dummy variable for whether IATTC waters were closed to vessels longer than 24m, Monthly El Nino indicator, Monthly El Nino indicator lagged by one year, Monthly El Nino indicator lagged by two years, and Monthly El Nino indicator lagged by three years. *p<0.1; **p<0.05; ***p<0.01

Correlation of Bigeye and Yellowfin Tuna Catch Across Fisheries

In Supplementary Figure 4, we plot a time series of annual bigeye and yellowfin tuna catch per 1,000 hooks for the Hawaii-based tuna fleet and the Hawaii-based swordfish fleet (we have a much longer time series here; the missing observations are due to the temporary closure of the swordfish fishery). The similarity in trends of CPUE is evident. The Pearson correlation coefficient for the two time series is 0.7. In Supplementary Figure 5, we plot a time series of annual bigeye and yellowfin tuna catch per 1,000 hooks for the Hawaii-based tuna fleet and the American Samoa-based fleet (which primarily targets albacore and yellowfin tuna). The similarity in trends of CPUE is also evident. The Pearson correlation coefficient for the two time series is 0.76.

Supplementary Figure 4: Correlation Between Hawaii Tuna Fleet and Hawaii Swordfish Catch of Bigeye and Yellowfin Tuna

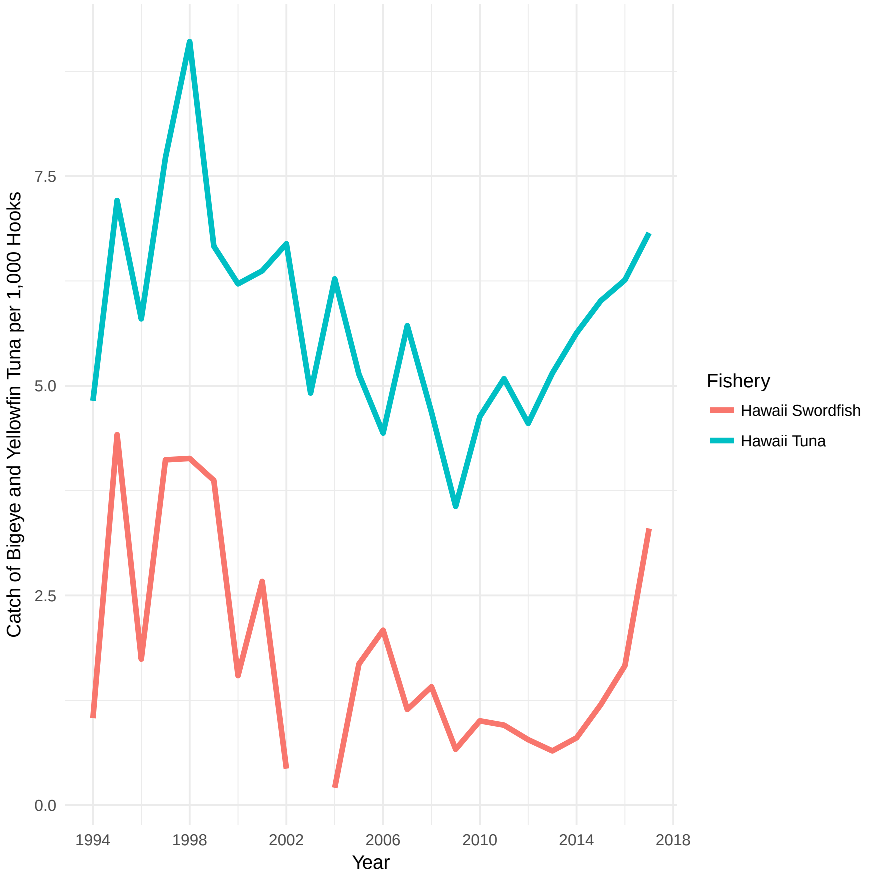

Supplementary Figure 5: Correlation Between Hawaii and American Samoa Catch of Bigeye and Yellowfin Tuna

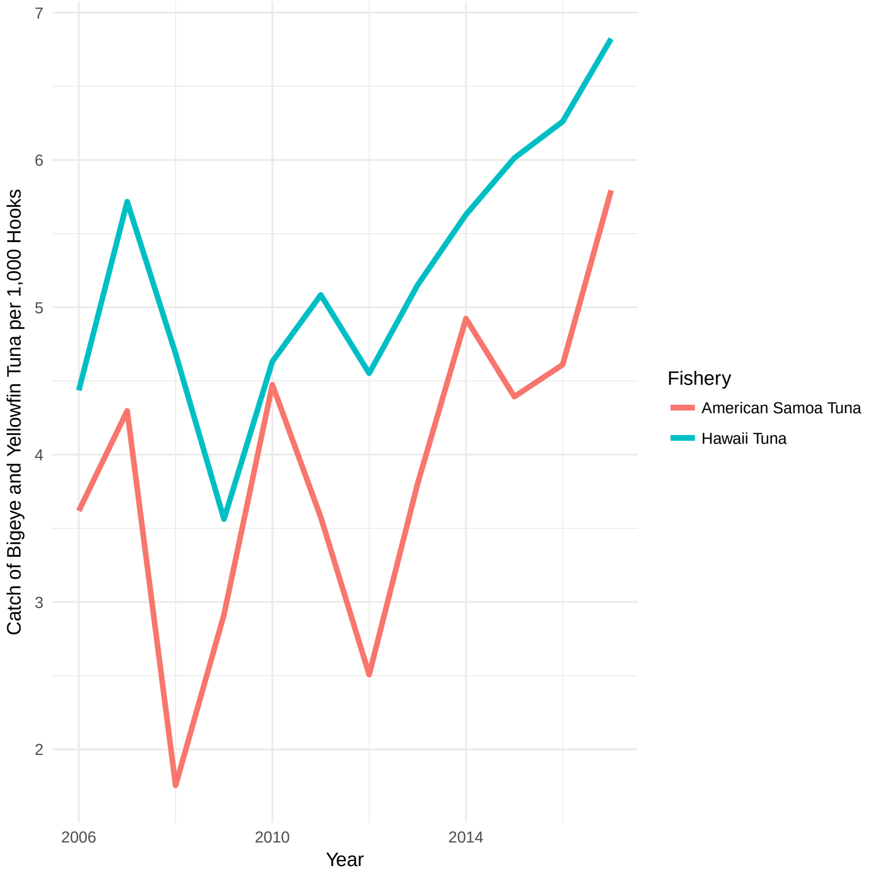

Testing the No Interference Assumption

The most obvious mechanism through which interference could take place is if the expansions moved treated fishers into the fishing areas of control fishers, thereby influencing their productivity through congestion, information sharing, localized depletion, etc.[6] We test for whether there is any evidence that interference is taking place using a series of simple tests. We calculate the average distance between the treated and untreated fleets by month and then test whether this decreases following either expansion. The results of regressing distance on a dummy variable for the expansion (and dummy variables for each month) are shown in Supplementary Table 7. We do not find any evidence of decreases in average distance and, in some cases, observe statistically significant increases in distance apart following the expansions. The intercept coefficients in the regressions in Supplementary Table 7 help to explain why interference does not seem to be a concern. For PRI, the average distance between the treated and untreated fleets prior to either expansion exceeds 1,700km and for PMNM it is larger than 4,000km (which is roughly the distance between Hawaii and American Samoa).

Supplementary Table 7: Congestion/Interference Estimation

	(1)	(2)	(3)	(4)
Constant	1,718.429***	2,175.581***	4,102.583***	4,230.098***
	(100.494)	(145.093)	(73.438)	(107.891)
PRI Expansion	198.733	151.388		
	(188.132)	(105.203)		
PMNM Expansion			273.733**	246.411*
			(111.005)	(132.819)
Month Dummies	No	Yes	No	Yes
Observations	76	76	93	93
R^2	0.015	0.739	0.025	0.677

Notes: The dependent variable is Average Monthly Distance (km) Between Treatment and Control Fleets. In Columns (1) and (2) the sample runs from January 2010 to August 2016. In Columns (3) and (4) the sample runs from January 2010 to December 2017. Heteroskedasticity-robust standard errors presented in parentheses. *p<0.1; **p<0.05; ***p<0.01

Difference-in-Differences Estimates for the Three Other Measures of Catch-per-Unit-Effort

Supplementary Tables 8, 9, and 10 present the same information as the main table in the paper except using different dependent variables. The effect of the expansions on CPUE is always positive or statistically indistinguishable from zero, except in one specification: Column (6) of Supplementary Table 9. This specification includes a suite of oceanographic controls and is also a measure of CPUE susceptible to bias since the effort exerted on one "trip" could vary dramatically from boat to boat, year to year, and across fisheries.

Supplementary Table 8: Difference-in-Differences Estimation of Catch per Set

	Dependent variable:					
	Catch of Bigeye and Yellowfin Tuna per Fishing Set					
	(1)	(2)	(3)	(4)	(5)	(6)
PRI Expansion	0.435***	−2.047***	−2.226***			
	(0.049)	(0.383)	(0.410)			
PMNM Expansion				2.826***	−1.245	−1.557
				(0.821)	(0.880)	(1.075)
Hawaii-based Tuna Trips	10.791***	10.833***	10.816***	1.339***	0.876***	3.751***
	(0.095)	(0.105)	(0.226)	(0.226)	(0.231)	(0.981)
PRI * Hawaii	2.967***	3.171***	2.660***			
	(0.180)	(0.184)	(0.224)			
PMNM * Hawaii				2.391***	2.658***	1.907**
				(0.847)	(0.851)	(0.922)
Month Dummies	No	Yes	Yes	No	Yes	Yes
Year Dummies	No	Yes	Yes	No	Yes	Yes
Vessel Dummies	No	No	Yes	No	No	Yes
Additional Controls	No	No	Yes	No	No	Yes
Observations	33,444	33,444	33,444	34,964	34,964	34,964
R^2	0.200	0.214	0.259	0.022	0.044	0.099

Notes: In Columns (1)-(3), the control group is Hawaii-based swordfish trips and the sample runs from January 1st 2010 to August 25th 2016. In Columns (4)-(6), the control group is American Samoa-based tuna trips and the sample runs from January 1st 2010 to December 31st 2017. Heteroskedasticity-robust standard errors presented in parentheses. The Additional Controls are whether the set included an experimental component, a dummy variable for whether the WCPFC waters were closed to fishing, a dummy variable for whether IATTC waters were closed to vessels longer than 24m, Monthly El Nino indicator, Monthly El Nino indicator lagged by one year, Monthly El Nino indicator lagged by two years, and Monthly El Nino indicator lagged by three years. *p<0.1; **p<0.05; ***p<0.01

Supplementary Table 9: Difference-in-Differences Estimation of Catch per Trip

	Dependent variable:					
	Catch of Bigeye and Yellowfin Tuna per Fishing Trip					
	(1)	(2)	(3)	(4)	(5)	(6)
PRI Expansion	7.317***	−33.199***	−37.707***			
	(2.153)	(11.749)	(11.222)			
PMNM Expansion				196.504**	168.002*	171.894**
				(88.522)	(91.007)	(68.056)
Hawaii-based Tuna Trips	147.018***	143.948***	165.090***	−141.289***	−147.945***	−89.937
	(2.990)	(3.390)	(6.989)	(25.321)	(25.591)	(74.273)
PRI * Hawaii	30.526***	34.478***	25.664***			
	(5.495)	(5.738)	(6.648)			
PMNM * Hawaii				−137.863	−131.283	−148.036**
				(88.761)	(89.976)	(64.120)
Month Dummies	No	Yes	Yes	No	Yes	Yes
Year Dummies	No	Yes	Yes	No	Yes	Yes
Vessel Dummies	No	No	Yes	No	No	Yes
Additional Controls	No	No	Yes	No	No	Yes
Observations	2,284	2,284	2,284	2,338	2,338	2,338
R^2	0.355	0.389	0.559	0.111	0.152	0.419

Notes: In Columns (1)-(3), the control group is Hawaii-based swordfish trips and the sample runs from January 1st 2010 to August 25th 2016. In Columns (4)-(6), the control group is American Samoa-based tuna trips and the sample runs from January 1st 2010 to December 31st 2017. Heteroskedasticity-robust standard errors presented in parentheses. The Additional Controls are whether the set included an experimental component, a dummy variable for whether the WCPFC waters were closed to fishing, a dummy variable for whether IATTC waters were closed to vessels longer than 24m, Monthly El Nino indicator, Monthly El Nino indicator lagged by one year, Monthly El Nino indicator lagged by two years, and Monthly El Nino indicator lagged by three years. *p<0.1; **p<0.05; ***p<0.01

Supplementary Table 10: Difference-in-Differences Estimation of Catch per Kilometer Traveled

	Dependent variable:					
	Catch of Bigeye and Yellowfin Tuna per Kilometer Traveled					
	(1)	(2)	(3)	(4)	(5)	(6)
PRI Expansion	0.001^{**}	-0.014^{*}	-0.021^{***}			
	(0.001)	(0.008)	(0.008)			
PMNM Expansion				0.145^{*}	0.106	0.055
				(0.075)	(0.076)	(0.087)
Hawaii-based Tuna Trips	0.067^{***}	0.069^{***}	0.068^{***}	-0.124^{***}	-0.127^{***}	-0.054
	(0.002)	(0.002)	(0.003)	(0.019)	(0.020)	(0.035)
PRI * Hawaii	0.021^{***}	0.021^{***}	0.019^{***}			
	(0.003)	(0.004)	(0.004)			
PMNM * Hawaii				-0.112	-0.105	-0.086
				(0.076)	(0.076)	(0.083)
Month Dummies	No	Yes	Yes	No	Yes	Yes
Year Dummies	No	Yes	Yes	No	Yes	Yes
Vessel Dummies	No	No	Yes	No	No	Yes
Additional Controls	No	No	Yes	No	No	Yes
Observations	2,284	2,284	2,284	2,338	2,338	2,338
R^2	0.255	0.364	0.469	0.148	0.207	0.298

Notes: In Columns (1)-(3), the control group is Hawaii-based swordfish trips and the sample runs from January 1st 2010 to August 25th 2016. In Columns (4)-(6), the control group is American Samoa-based tuna trips and the sample runs from January 1st 2010 to December 31st 2017. Heteroskedasticity-robust standard errors presented in parentheses. The Additional Controls are whether the set included an experimental component, a dummy variable for whether the WCPFC waters were closed to fishing, a dummy variable for whether IATTC waters were closed to vessels longer than 24m, Monthly El Nino indicator, Monthly El Nino indicator lagged by one year, Monthly El Nino indicator lagged by two years, and Monthly El Nino indicator lagged by three years. $^{*}p<0.1$; $^{**}p<0.05$; $^{***}p<0.01$

Exploring the Catch per Trip Results

Since the decrease in catch-per-fishing-trip following the PMNM expansion appears to contradict the increases in catch-per-1,000-hooks and catch-per-fishing-set, we decided to explore this result in more detail. In particular, we wish to test whether the total amount of fishing effort exerted per trip is perhaps changing following the PMNM expansion. To do this, we replace catch-per-fishing-trip as the outcome variable in the regression in Column (6) of Supplementary Table 9 with three other measures of fishing effort per trip: total hooks per trip, total fishing sets per trip, and total number of days at sea (approximated as the number of days between the first and last set of the trip). It can be seen in Supplementary Table 11 that all three measures of fishing effort per trip decline in the Hawaii tuna fleet compared to the American Samoa tuna fleet. This explains the anomalous result for catch-per-fishing trip following the PMNM expansion.

Supplementary Table 11: Difference-in-Differences: Fishing Effort per Trip

	Dependent variable:		
	Total Hooks	Total Sets	Days at Sea
	(1)	(2)	(3)
Constant	55,012.250	5.357	22.137
	(42,278.460)	(15.363)	(18.125)
PMNM Expansion	21,603.250**	9.153***	11.659***
	(9,833.173)	(3.226)	(4.223)
Hawaii-based Tuna Trips	−41,416.500***	−12.173***	−16.083***
	(11,626.040)	(3.655)	(4.657)
PMNM * Hawaii	−19,984.170**	−9.311***	−10.330**
	(9,522.831)	(3.120)	(4.085)
Month Dummies	Yes	Yes	Yes
Vessel Dummies	Yes	Yes	Yes
Additional Controls	Yes	Yes	Yes
Observations	2,338	2,338	2,338
R^2	0.657	0.589	0.582

Notes: The dependent variable in Column (1) is total hooks deployed per trip. In Column (2) it is total sets per trip and in Column (3) it is the length of the trip in days. The sample runs from January 1st 2010 to December 31st 2017. Heteroskedasticity-robust standard errors presented in parentheses. The Additional Controls are whether the set included an experimental component, a dummy variable for whether the WCPFC waters were closed to fishing, a dummy variable for whether IATTC waters were closed to vessels longer than 24m, Monthly El Nino indicator, Monthly El Nino indicator lagged by one year, Monthly El Nino indicator lagged by two years, and Monthly El Nino indicator lagged by three years. *p<0.1; **p<0.05; ***p<0.01

Difference-in-Differences Estimates for Distance Traveled

In Supplementary Table 12, we expand our analysis of distance traveled using the previously explained Difference-in-Differences approach. We observe no evidence of an increase in distance traveled following either expansion.

Supplementary Table 12: Difference-in-Differences Estimation of Distance Traveled

	Dependent variable:					
	Trip Distance (km)					
	(1)	(2)	(3)	(4)	(5)	(6)
PRI Expansion	907.824***	822.958***	806.600***			
	(209.506)	(263.136)	(247.121)			
PMNM Expansion				−201.770	175.117	834.163***
				(231.485)	(289.042)	(293.568)
Hawaii-based Tuna Trips	−1,654.195***	−1,912.442***	−1,213.398***	718.725***	722.740***	−352.465
	(88.858)	(94.208)	(113.696)	(141.574)	(145.718)	(620.564)
PRI * Hawaii	−1,068.310***	−945.833***	−790.420***			
	(217.145)	(210.083)	(211.553)			
PMNM * Hawaii				36.022	−46.434	−356.455
				(239.624)	(278.134)	(262.731)
Month Dummies	No	Yes	Yes	No	Yes	Yes
Year Dummies	No	Yes	Yes	No	Yes	Yes
Vessel Dummies	No	No	Yes	No	No	Yes
Additional Controls	No	No	Yes	No	No	Yes
Observations	2,284	2,284	2,284	2,338	2,338	2,338
R^2	0.296	0.355	0.547	0.030	0.166	0.468

Notes: In Columns (1)-(3), the control group is Hawaii-based swordfish trips and the sample runs from January 1st 2010 to August 25th 2016. In Columns (4)-(6), the control group is American Samoa-based tuna trips and the sample runs from January 1st 2010 to December 31st 2017. Heteroskedasticity-robust standard errors presented in parentheses. The Additional Controls are whether the set included an experimental component, a dummy variable for whether the WCPFC waters were closed to fishing, a dummy variable for whether IATTC waters were closed to vessels longer than 24m, Monthly El Nino indicator, Monthly El Nino indicator lagged by one year, Monthly El Nino indicator lagged by two years, and Monthly El Nino indicator lagged by three years. *p<0.1; **p<0.05; ***p<0.01

GFW only has partial coverage of the fleet

One obvious critique of our analysis of trip distance using observer data is that we are missing the actual distance traveled from port to set, between sets, and from final set to port. In particular, this may be higher following the monument expansions if vessels are forced to do more searching between fishing sets because they are unfamiliar with new fishing grounds, fish are less abundant outside the monuments, or there is increased competition with other vessels on the high seas. Some of this additional distance could be missing from our estimates of trip distance. In order to test whether our results on distance are robust to using more fine-scale spatial information, we make use of a newly available dataset on fishing vessel locations provided by Global Fishing Watch.

Global Fishing Watch is a non-profit organization and website launched in September 2016 by Google in partnership with Oceana and SkyTruth to provide the world's first global view of commercial fishing activities. At any moment, as many as 200,000 vessels are publicizing their locations via a system known as the Automatic Identification System (AIS). AIS is intended, primarily, to allow ships to view marine traffic in their area and to be seen by that traffic. This requires a dedicated VHF (Very High Frequency) AIS transceiver that automatically broadcasts information about a vessel, such as its position, speed, navigational status, name, and VHF call sign. This information is broadcast at regular intervals, in some cases, as frequently as every fifteen seconds. Vessels fitted with AIS transceivers can be tracked by other ships, by AIS base stations located along coast lines or, when out of range of terrestrial networks, by a growing number of satellites that are fitted with special AIS receivers. The International Maritime Organization's International Convention for the Safety of Life at Sea requires AIS to be fitted aboard international voyaging ships with 300 or more gross tonnage, and for all passenger ships regardless of size. The US Coast Guard now requires it for all vessels larger than 65 feet. Despite its widespread use, AIS information typically supplements marine radar, which continues to be the primary method of collision avoidance for water transport.

Global Fishing Watch extracts the AIS tracks for fishing vessels and enables users with Internet access to monitor fishing activity globally, and to view individual vessel tracks. They also partner with

Supplementary Figure 6: Number of Vessels Transmitting an AIS Signal by Month

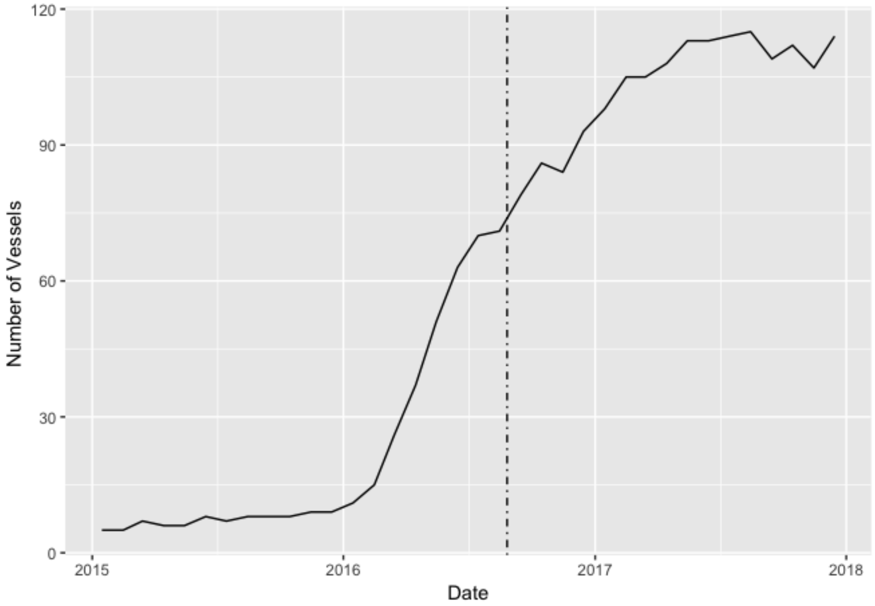

Notes: Vertical dashed line indicates the date of the Papahānaumokuākea expansion: August 26, 2016.

academic researchers to provide more fine-scale data. We requested and obtained individual fishing tracks for all of the vessels that we could identify within the Hawaii and American Samoa longline fisheries. We obtained tracks for 148 different vessels (identified by their MMSI: Maritime Mobile Service Identity number) but a number of these vessels had yet to emit a signal leaving a total of 128 vessels with observed tracks. Initially, the dataset contains 5,592,202 observations of vessel locations ranging from January 1st 2015 to December 31st 2017.

Unfortunately, the GFW dataset for both longline fisheries is quite incomplete. This is because vessels in the Hawaii and American Samoa longline fisheries were not required to have AIS transceivers on-board until very recently. On March 1st 2016, the United States Coast Guard introduced a mandate requiring AIS on all US vessels larger than 65 feet, which includes all of the pelagic longline vessels in Hawaii and some of the larger vessels in American Samoa (https://www.navcen.uscg.gov/?pageName=AISRequirementsRev). However, most Hawaii-based vessels appear to have initially ignored this mandate and not obtained an AIS transceiver or switched it on until late 2016. Supplementary Figure 6 shows the number of vessels emitting an AIS signal by month from 2015 to 2017. It can be clearly seen that we only have partial coverage of the fleet prior to the Papahānaumokuākea expansion, which is indicated by a vertical dashed line.

For 2017, there appears to be very strong overlap between the GFW dataset and logbook records. According to official NOAA records, there were 145 longline vessels active in 2017. Of these, 129 were identified to have been broadcasting AIS. The AIS data tracks 25,862 days at sea, compared with 27,716 reported by NOAA logbook data, suggesting that for vessels broadcasting AIS, more than 90 percent of the days at sea are being recorded. This number may also undercount the amount of time broadcasting, as some vessels acquired AIS partway through the year, meaning that the missing days may be because the vessels did not yet have AIS, not because they were failing to turn them on. However, prior to 2017, the coverage is very patchy. In the years before 2017, the AIS data has much lower coverage. In 2016, 102 vessels broadcast, and only about half of the official logbook activity was captured, likely because many of these vessels acquired AIS partway through the year. In 2015, before the Coast Guard requirement, the coverage was much lower - only 6 Hawaii longline vessels broadcast an AIS signal in 2015. In 2012, it was less than four vessels. Thus, we proceed with caution

Supplementary Figure 7: Monthly Distance Traveled for Vessels with the Longest Time Series in the GFW Database

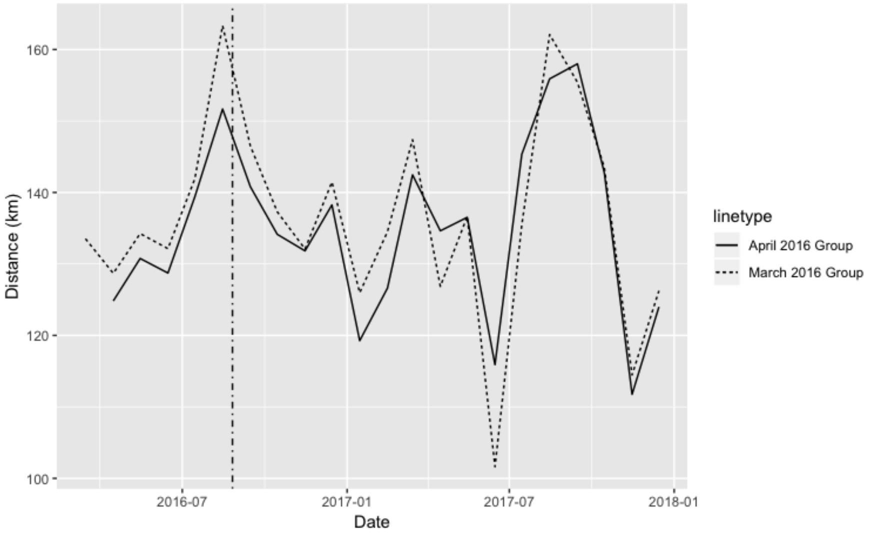

Notes: Vertical dashed line indicates the date of the Papahānaumokuākea expansion: August 26, 2016.

in interpreting our findings but can at least hope to see whether trends in this fine-scale but incomplete dataset match those in the more aggregated but representative observer data. Supplementary Figure 7 plots the mean distance travelled by month for two different groups of vessels. The first group is all vessels that broadcast an AIS signal in March 2016 (26 vessels). The second group is all vessels that broadcast an AIS signal in April 2016 (37 vessels).

Regression-based Analysis of Daily Distance Traveled Using GFW data

We perform regression-based analysis in Supplementary Table 13 using the same regression model as in Equation 1 except that we do not include a PRI dummy. In all three columns, the dependent variable is the daily distance traveled. Column (1) has no controls, Column (2) includes vessel fixed effects, and Column (3) includes vessel and month fixed effects. In Column (1) we observe an increase in distance traveled but this could simply be due to more and more vessels turning on their transceivers after the expansion. Once we control for individual vessel differences in Columns (2) and (3), we fail to reject the null hypothesis that distance traveled has not increased since the monument was expanded. In fact, in Column (2), we can reject the null that the increase in distance traveled per day was greater than 12km (an 8% increase) at the 5% level of statistical significance. In summary, the GFW data does not support the argument that the fleet is traveling further following the Papahānaumokuākea expansion.

Supplementary Table 13: Distance Traveled

	(1) Distance	(2) Distance	(3) Distance
PMNM Expansion	7.022***	0.947	6.620
	(1.418)	(5.519)	(6.329)
Constant	137.0***	142.1***	123.2***
	(1.312)	(4.683)	(7.982)
Vessel Fixed Effects	No	Yes	Yes
Month Dummies	No	No	Yes
Observations	48977	48977	48977

Standard errors in parentheses

* $p < 0.05$, ** $p < 0.01$, *** $p < 0.001$

Bias Estimates

A further concern with our distance estimates is that the bias or missing distance may have increased following the expansions. This would lead us to falsely conclude that distance traveled has not increased when in fact it has. We address this by identifying all complete trips that have both observer and GFW data indicating the length of the trip. Unfortunately, this only leaves a small number of trips prior to the Papahānaumokuākea expansion. We are able to identify 19 trips before and 184 trips after the expansion. The bias before is -1,414 km and after it is -1,451 km (meaning that our observer-based measure of estimated trip distance underestimates the true trip distance). The relative bias is -26% before and -34% after, with an overall bias of 33% less than the true trip distance. Neither of these before-after differences are statistically significant when we regress the bias on a dummy variable for the PMNM expansion (see Supplementary Table 14). Thus, we do not observe evidence that the mis-measurement of trip distance using our set-based approach has increased following the Papahānaumokuākea expansion.

Supplementary Table 14: Bias Regressions

	Dependent variable:	
	Absolute Bias	Relative Bias
	(1)	(2)
Constant	−1,413.621***	−0.260***
	(249.803)	(0.054)
PMNM Expansion	−37.528	−0.082
	(259.429)	(0.058)
Observations	203	203
R^2	0.0001	0.007

Notes: In Column (1), the dependent variable is the absolute bias and in Column (2) it is the relative bias. The sample runs from April 2016 to December 2017. Heteroskedasticity-robust standard errors presented in parentheses. *p<0.1; **p<0.05; ***p<0.01

Supplementary References

[1] Rieser, A. & Van Dyke, J. M. New marine national monuments settle issues. *Nat. Res. & Env.* **24**, 50 (2009).

[2] Barnes, M. L., Lynham, J., Kalberg, K. & Leung, P. Social networks and environmental outcomes. *Proc. Natl. Acad. Sci. USA* **113**, 6466–6471 (2016).

[3] Pan, M. Economic characteristics and management challenges of the Hawaii pelagic longline fisheries: Will a catch share program help? *Mar. Policy* **44**, 18–26 (2014).

[4] Ayers, A. L., Hospital, J. & Boggs, C. Bigeye tuna catch limits lead to differential impacts for Hawaii longliners. *Mar. Policy* **94**, 93–105 (2018).

[5] Trenberth, K. The climate data guide: Niño SST indices (Niño 1+ 2, 3, 3.4, 4; ONI and TNI). *National Center for Atmospheric Research: Boulder, CO, USA* (2016). URL https://climatedataguide.ucar.edu/climate-data/nino-sst-indices-nino-12-3-34-4-oni-and-tni.

[6] Ferraro, P. J., Sanchirico, J. N. & Smith, M. D. Causal inference in coupled human and natural systems. *Proc. Natl. Acad. Sci.* **116**, 5311–5318 (2019).
